# RNA-binding protein 39: a promising therapeutic target for cancer

**DOI:** 10.1038/s41420-021-00598-7

**Published:** 2021-08-13

**Authors:** Caipeng Xu, Xiaohua Chen, Xuetian Zhang, Dapeng Zhao, Zhihui Dou, Xiaodong Xie, Hongyan Li, Hongying Yang, Qiang Li, Hong Zhang, Cuixia Di

**Affiliations:** 1 Bio-Medical Research Canter, Institute of Modern Physics, Chinese Academy of Sciences, Lanzhou, 730000 China; 2 Key Laboratory of Heavy Ion Radiation Biology and Medicine of Chinese Academy of Sciences, Lanzhou, 730000 China; 3 College of Life Sciences, University of Chinese Academy of Sciences, Beijing, 101408 China; 4 School of Nuclear Science and Technology, University of Chinese Academy of Sciences, Beijing, 101408 China; 5 School of Basic Medical Sciences, Lanzhou University, Lanzhou, 730000 China; 6 School of Radiation Medicine and Protection, Medical College of Soochow, Soochow, 215006 China; 7 Advanced Energy Science and Technology Guangdong Laboratory, Huizhou, 516029 China

**Keywords:** Cancer therapy, Predictive markers

## Abstract

RNA-binding motif protein 39 (RBM39), as a key factor in tumor-targeted mRNA and protein expression, not only plays a vital role in tumorigenesis, but also has broad development prospects in clinical treatment and drug research. Moreover, since RBM39 was identified as a target of sulfonamides, it has played a key role in the emerging field of molecule drug development. Hence, it is of great significance to study the interaction between RBM39 and tumors and the clinical application of drug-targeted therapy. In this paper, we describe the possible multi-level regulation of RBM39, including gene transcription, protein translation, and alternative splicing. Importantly, the molecular function of RBM39 as an important splicing factor in most common tumors is systematically outlined. Furthermore, we briefly introduce RBM39’s tumor-targeted drug research and its clinical application, hoping to give reference significance for the molecular mechanism of RBM39 in tumors, and provide reliable ideas for in-depth research for future therapeutic strategies.

## Facts

- RBM39 is characterized by upregulated expression in many cancers, but the precise molecular function in specific cancers may not be thorough enough.
- RBM39 is indirectly involved in tumor growth and progression by regulating transcription of many tumor-related genes, protein translation, and alternative splicing.
- RBM39 can be exploited as markers for more accurate diagnosis, as well as improved prognosis and monitoring of treatment response in patients with cancer.
- RBM39 is identified as a target of sulfonamides, and it has played a key role in the emerging field of small molecule drug development.

## Open questions

- What is the exact molecular function of RBM39 in normal and cancer tissues?
- What is the preferential effect of RBM39 degradation in cancer cells?
- Would SPLAMs be used in more research tests and clinical patient treatments to improve the potential clinical efficacy of SPLAMs?
- Would SPLAM derivatives recruit new substrates other than RBM39 to join CUL4–DCAF15 to better exert the clinical value of the drug?

## Introduction

Cancer has always been regarded as a highly complex disease, which seriously threatens human health and life. People have been committed to the treatment and clinical research of cancer for a long time, so it is necessary to deeply understand the molecular mechanism of cancer progression and research on targeted drugs. Here, RNA-binding motif protein 39 (RBM39), also known as splicing factor HCC1, CAPERα, FSAP59, RNPC2, and CAPER alpha, is an essential serine/arginine-rich (SR) RNA-binding protein, as well as a pre-mRNA splicing factor and transcription coactivator. RBM39 was initially identified as the autoantigen from a patient with liver cirrhosis who later developed hepatocellular carcinoma [1]. With in-depth research on the characteristics and multiple functions of RBM39, it is revealed that RBM39 is mainly involved in biological processes, such as transcriptional regulation, alternative splicing, and protein translation [2]. An increasing body of data suggests that RBM39 has been closely linked to malignant progression of various cancers, and its expression may be related to RBM39 mutual proteins in specific environments. Notably, RBM39 is upregulated in most cancers, and the inhibition of its function is lethal to several cancers including lung cancer [3], breast cancer [4], and colorectal cancer [5]. For instance, reducing the expression of RBM39 can inhibit the proliferation of estrogen receptor (ER)-positive human breast cancer cells [6, 7], which further suggests that RBM39 is an important signaling molecule in the pathogenesis of breast cancer, which may bring new methods for treatment of invasive cancer [4]. Interestingly, RBM39 is a potential tumor suppressor in few human malignancies. RBM39 could effectively recede the carcinogenic activity of NF-κb-Rel protein in lymphocytes [8], and its overexpression inhibited tumor angiogenesis and growth [9]. Therefore, small-molecule drugs targeting RBM39 may be an extremely valuable method in the current treatment of various malignant tumors.

In recent years, although there have been relevant reports on the research of RBM39 in tumorigenesis and development, the precise function in specific cancers may not be thorough enough. In addition, due to the limited efficacy of sulfonamides, its research progress in clinical trials is relatively slow, so it is very important to try to reveal its latest research progress in tumors. In this review, we mainly summarized the possible regulation mechanism of RBM39 involved in tumor, and briefly discussed the molecular function of RBM39 in tumors. Furthermore, we outlined the emerging tumor-targeted drug research and its clinical application. Thus, it is highlighting the significance of RBM39 as targets for cancer therapy.

## Characteristics of RBM39

RBM39 has significant sequence similarity with splicing factors U2AF65, and RBM23 (Table 1), and contains both R–X–F elements and U2AF homology motif (UHM) negatively charged α-helix A [1, 2, 10]. It is similar to RBM23 and U2AF65 shares the N-terminal RS domain together with RBM23 and PUF60, and shares RRM1/RRM2 and C-terminal UHM [11]. In particularly, U2AF65 contains the U2AF ligand substrate, others do not [12] (Table 1A). HCC1/RBM39 contains two alternately spliced subtypes, called HCC1.3 and HCC1.4. HCC1.4, a full-length cDNA clone encoding 530 amino acids, can interact with the C-terminus of the SR-related protein SRrp53, and SRrp53 has been shown to activate the 3′weak splice site [13]. HCC1.3 is another representative clone of RBM39, and its 18 nucleotide (six amino acids) deletion is located in the third RNA-recognition motif (RRM) domain, which can affect the splicing reaction [1, 14, 15]. It has been revealed that HCC1.4 and HCC1.3 as TAAs have different autoantibody immune responses [3], but they are not distinguishable in terms of binding and transcriptional coactivation properties. Additionally, RBM39 mRNA exhibitings obvious tissue specificity in normal tissues has been discovered. For example, it is highly transcribed in CD56 + natural killer cells, CD4 + and CD8 + T cells, CD19 + B lymphocytes, CD34 + cells and CD33 + myeloid cells, and is expressed in large amounts in immune system-related cells, lymph-node cells, uterine cells, and thyroid cells [16]. In brief, the characteristics and tissue specificity of RBM39 determine that it may be involved in a variety of biological processes.

**Table 1:** Research application of RBM39 in tumor.

Cancer	Expression	Related Function	Refs
Ewing Sarcoma	Up	Regulates proliferation and protein synthesis pathways	[1]
Breast cancer	Up	Mediates VEGF alternative splicing	[2]
TNBC	Up	Regulates DNA repair, cell apoptosis and cell cycle	[3, 4]
Invasive ductal carcinoma	Up	Transfers from the cytoplasm to the nucleus	[5]
Colorectal adenoma	Up	Mediates cell viability	[6]
	Up	Affects cell survival and viability	[7]
Hepatocellular carcinoma	Up	Related to the appearance of microvessels	[8]
Prostate cancer	Down	Cancer cell migration and invasion	[9]
Myeloma malignancy	Up	Regulates mTOR signaling, promotes cell proliferation	[10]
AML	Up	Causes changes in the splicing of HOXA9 target genes	[11]
Lung cancer	Up	Promotes proliferation and migration	[12]

**Table 2:** Clinical trials of aryl sulfonamides.

Type	NCT Number	Condition	Phase	Start Date
Indisulam	NCT00165594	Gastric cancer	Phase 1, Phase 2	February 2005
	NCT00165867	Colorectal cancer	Phase 2	April 2005
	NCT01692197	Leukemia	Phase 2	February 2013
	NCT00059735	Kidney Neoplasms, Carcinoma, Adenocarcinoma,	Phase 2	May 2002
	NCT00165880	Breast Cancer	Phase 2	December 2004
	NCT00165854	Colorectal cancer	Phase 2	March 2003
	NCT00014625	Melanoma (Skin)	Phase 2	February 2001
	NCT00080197	Breast neoplasms	Phase 2	February 2004
E7820	NCT01133990	Colorectal cancer	Phase 1, Phase 2	April 2010
	NCT00309179	Advanced colorectal cancer	Phase 2	September 2007
	NCT00078637	Malignant neoplasms, lymphoma	Phase 1	January 2004
	NCT01347645	Colon cancer, Rectal cancer	Phase 1, Phase 2	September 2011
	NCT01773421	Advanced solid tumors	Phase 1	June 2011
CQSC	NCT00005864	Colorectal cancer	Phase 2	April 2000
	NCT00008372	Lung cancer	Phase 2	December 2000
Tasisulam	NCT01215916	Solid tumors	Phase 1	February 2008
	NCT01258348	Metastatic renal cell cancer	Phase 1	July 2008
	NCT01214668	Solid tumors	Phase 1	January 2009
	NCT00383292	Metastatic melanoma	Phase 2	November 2006
	NCT00992225	Breast cancer	Phase 2	September 2009
	NCT00363766	Non-small-cell lung Cancer	Phase 2	September 2006
	NCT01284335	Advanced solid tumors	Phase 1	July 2008
	NCT01006252	Melanoma	Phase 3	December 2009
	NCT00718159	Acute myeloid leukemia	Phase 1	August 2008
	NCT01185548	Lymphoma, Advanced cancer	Phase 1	July 2010
	NCT00490451	Sarcoma, Soft tissue	Phase 2	August 2007
	NCT00428610	Ovarian, Fallopian tube, Primary peritoneal cancer	Phase 2	February 2007

## Regulation mechanism of RBM39

### RBM39 coordinates transcriptional regulation

**Fig. 1:**
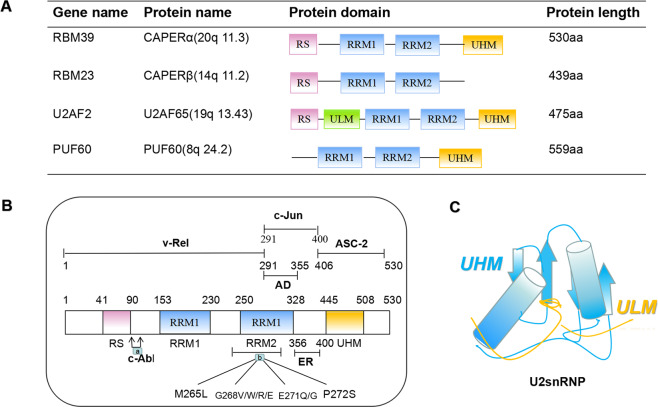
The domain of RBM39 and its related proteins. **A** The domain composition of U2AF65 and its paralogous proteins**. B** Different functional domains of RBM39 (a. The interaction between c-Abl and RBM39, the phosphorylation site of RBM39; b. The toxic effect of indisulam, the mutation domain of indisulam gathers in the RRM2 domain of RBM39). **C** Schematic of the UHM–ULM-interaction structure.

**Fig. 2:**
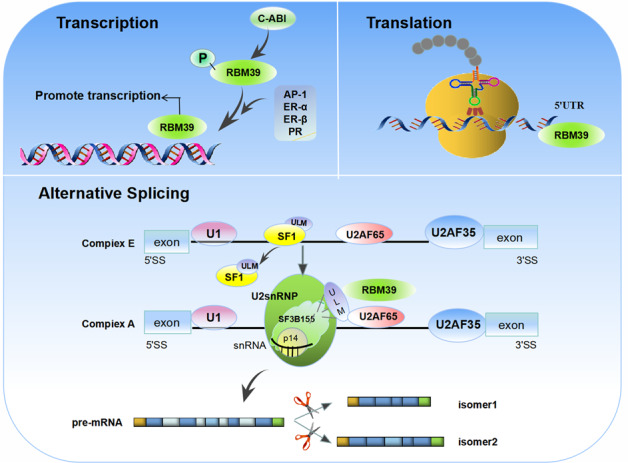
Schematic diagram of the function of RBM39 in cancer cells. RBM39 participates in the regulation of gene expression from multiple levels, including transcription, alternative splicing, and translation.

It has been previously demonstrated that RBM39 is involved in transcriptional regulation of related proteins and various genes (Fig. 1B, Fig. 2). RBM39 can bind to the transcription factor activating protein-1 (AP-1)/c-Jun and the nuclear steroid receptors ER-α and ER-β in vivo and effectively promote its transcription [2, 14, 17]. In addition, it interacts with ASC-2, a coactivator that may act to recruit molecules containing RRM domains (Fig. 1B). Dowhan et al. pointed out that RBM39 could interact with progesterone receptor (PR) in vitro and stimulate the transcriptional activity of hormone-mediated PR [2]. Interestingly, RBM39 had high selectivity for c-Jun, ERα/β, and PR, and did not have an effect on other transcription factors, such as p53, serum-response factor, c-fos, NF-κB component p50, glucocorticoid hormone receptor, farnesoid X receptor, thyroid hormone receptor-α/β, and liver X receptor-α/β, among others [2, 8, 14]. It is reported that RBM39 had higher transcript levels in brain cancer and ovarian cancer [18]. Additionally, the study revealed that a total of 304 differentially expressed genes in RBM39-deficient cells were significantly regulated by RBM39, of which 123 were downregulated and 181 were upregulated, indicating that RBM39 could affect the regulation of transcription levels [19]. Furthermore, Mai et al. investigated that as a substrate of tyrosine kinase, RBM39 had a structural interaction with c-Abl [20]. Importantly, c-Abl could phosphorylate RBM39 and promote the transcriptional coactivation activity of RBM39 for ERα and PRβ, making it highly expressed in the placenta and liver tissues of the steroid hormone-dependent signaling pathway [20]. It is considered that RBM39 induced nuclear genes by coactivating ERR-α-Gabpa, which could directly lead to transcriptional regulation of regulators, including C-MYC and Gabpa [21]. Kumar et al. revealed that RBM39 could interact with TBX3 to inhibit the transcription of CDKN2A-p16^INK^ and Rb pathways. Therefore, RBM39/TBX3 and UCA1 constituted an important mechanism to regulate the transcription of CDKN2A-p16^INK^ [22]. In general, RBM39 enhances the activity of transcription factors and participates in the formation of the initiation complex through the interaction between transcription factors that bind to promoters [2].

### RBM39 regulates protein translation

It is demonstrated that RBM39 binds to mRNAs related to coding and translation proteins and participates in the expression of translation-related proteins [23], but there are currently not many such reports. Critically, the binding of RBM39 at the 5′UTR site could affect protein translation, whereas particularly under conditions of this special combination of 5′/3′ splice sites, it might be more conducive to selective splicing regulation [19]. Mai et al. found that the genes located at the binding peak of RBM39 were enriched in protein translation, and through in vitro translation experiments proved that the translation of luciferase mRNA was significantly inhibited after the RBM39 gene was knocked out [19]. Meanwhile, it is well established that the downregulation of RBM39 activity reduced the expression of cell cycle advancement regulators and inhibited protein-synthesis pathways, thereby affecting the protein-translation process [6]. In addition, current evidence suggests that RBM39 undergoes post-translational modifications, including ubiquitination and phosphorylation. Depending on this, RBM39 plays an extremely critical role in regulating protein translation in multiple cell processes.

### RBM39 regulates alternative splicing

Alternative splicing is a highly regulated and coordinated molecular mechanism involving multiple physiological processes [24]. RBM39 has been considered as a potential splicing factor, which plays an important role in pre-mRNA alternative splicing of human, yeast and Drosophila [25, 26], It could colocalize with splicing factors SRSF2, SC35, and m3G at the nuclear spot, which was rich in pre-mRNA splicing proteins [1, 15]. As the U2snRNP related splicing factor 3b subunit 155 (SF3b155) contains U2AF ligand motif (ULM), RBM39 could interact with U2AF65 [27, 28], SF3B155 [27, 28], and RSRC1 [13], which might provide an opportunity to regulate alternative splicing. RBM39 combined with essential splicing factors in specific complexes through UHM/ULM interaction, promoted the recruitment of U2snRNP and the coordination of splicing factors, so as to form splice complexes and splice pre-mRNA species. Therefore, RBM39 might interact with splicing factors related to early recognition of 3′splice sites, thereby affecting the progress of splicing reactions.

Alternative splicing of RBM39 is an extremely important mechanism, and its regulatory mechanism is similar to that of U2AF65, which can directly bind to RNA or recruit specific splicing factors to regulate selective splicing. It was further revealed that as many as 20% of alternative splicing exons were regulated by both RBM39 and U2AF65 [19]. Mai et al. studied RBM39-mediated alternative splicing in human breast cancer MCF-7 cells and found a total of 359 RBM39-regulated alternative splicing events, of which cassette exons were the most common regulatory target [19]. Moreover, genes regulated by RBM39-mediated alternative splicing were involved in G2/M cell cycle transition and cell response to DNA damage [6, 19]. Regarding the regulation of alternative splicing of RBM39, it was further confirmed that in T47D breast cancer cells, downregulated expression of RBM39 could correspondingly change the alternative splicing of vascular endothelial growth factor (VEGF) and increase the ratio of VEGF121 and VEGF189 in breast cancer [14]. Huang et al. demonstrated in Ewing cells that the overexpression of RBM39 mediated the alternative splicing of VEGF and controlled the transition from VEGF189 to VEGF165, while the downregulation of RBM39 led to an increase in the ratio of VEGF165/VEGF189 [9]. Subsequently, Taisuke et al. confirmed that VEGF189 mRNA was significantly decreased and VEGF121mRNA was increased after treatment with these sulfonamides or RBM39 siRNA [29]. The splicing regulator polypyrimidine tract-binding protein inhibited the excision of alternatively spliced exons by preventing U2AF from binding at the 3′splice site and 5′splice site dependently [30]. In addition, RBM39 promoted the inclusion of false exons in the iron–sulfur cluster assembly gene by inhibiting the binding to polypyrimidine tract-binding protein, thereby affecting the specific splicing selection of RBM39 [31]. Interestingly, RBM39 is also thought to play an important role in SIN3B alternative splicing. Studies identified that BMP4-dependent transcription was regulated by targeted negative feedback of SIN3B alternative splicing, which could enhance BMP activity after knocking out RBM39 [32]. Taken together, as a vital splicing factor, RBM39 participates in a wide range of alternative splicing of pre-mRNA. Importantly, based on its biological role and clinical significance in tumor progression, it might bring new ideas for elucidating the mechanism of tumor occurrence, development, and metastasis. Nevertheless, the extent to which RBM39 is involved in alternative splicing is still unknown, so it is important to explore the regulatory mechanism of RBM39 alternative splicing.

## Molecular function of RBM39 in cancer

### Breast cancer

A large number of data suggested that RBM39 regulated the transcriptional activity of ERs and PRs and was involved in the progression of human breast cancer [4, 6, 33]. RBM39 was expressed at high levels in human breast cancer tissues, but it was almost undetectable in normal breast tissues [6]. In addition, RBM39 was also overexpressed in mouse models of breast cancer and was associated with increased estrogenic sensitivity, indicating that it could play an important role in the early development of ER-positive breast cancer [6]. Mercier et al. found that RBM39 expression was positively correlated with tumor size in vitro and in vivo, and virus-mediated knockdown of RBM39 in ER-positive MCF-7 cells could significantly hinder the proliferation of human breast cancer cells and tumor growth in vivo [6]. To a certain extent, it might also inhibit AP-1/c-Jun transcriptional activity in MCF-7 cells, thereby inhibiting the phosphorylation of c-Jun [6, 34]. Regarding the mechanism, knockdown of RBM39 inhibited cell proliferation and protein-synthesis pathways [6]. Another study documented that the expression of RBM39 was significantly upregulated in human triple-negative breast cancer (TNBC) specimens compared with normal breast tissue. If the expression of RBM39 was inhibited, the proliferation of human breast cancer cells could be significantly reduced[4]. Furthermore, in terms of mechanism, knockdown of RBM39 could impair the functional repair of DNA by inhibiting the activity of RAD51, c-Abl and Rb protein-synthesis pathways, and then induce apoptosis. It can be seen that RBM39 is expected to become a potential therapeutic target and biomarker for TNBCs to predict the response to DNA damage treatment. Through the analysis and identification of human breast cancer samples, it was found that the overexpression of RBM39 experienced cytoplasmic-to-nuclear metastasis in the process of breast cancer metastasis from pre malignant to ductal carcinoma [35]. It follows that RBM39 has extremely broad development prospects in the treatment of this aggressive cancer.

### Acute myeloid leukemia

Acute myeloid leukemia (AML) is an aggressive hematological malignant tumor with a high recurrence rate after conventional combined chemotherapy [36]. Regarding the treatment of AML, one aspect was dedicated to mutation-specific targeted drug research [37], and the other was to identify other nonmutant proteins that were necessary for the survival of AML cells. Among them, H3b-8800 was one of these splicing factor inhibitors, which was currently in phase I clinical trial of refractory myeloid malignancies [38]. Proteomics studies found that there was an important RBP splicing network in AML, as a key member of the RBP. RBM39 was closely dependent on cancer [39]. In particular, degradation of RBM39 led to abnormal splicing of transcriptional regulators necessary for the survival of AML, thus playing an extremely critical role in maintaining RNA splicing and survival of AML. Recently, Wang et al. revealed that knocking out RBM39 could slow the progression of leukemia and improve overall survival. Notably, the anticancer effects of RBM39 gene knockout in indisulam treatment were studied in vitro and in vivo, and SPlicing inhibitor sulfonamides (SPLAMs) were found to be an effective and safe AML treatment with minimal adverse effects on hematopoietic cells [40]. Moreover, follow-up studies clarified the mechanism by which CRISPR-mediated degradation of RBM39 led to incorrect splicing of HOXA9 target genes and extensive antileukemia effects [41]. In general, RBM39 is critical for AML cell survival and disease progression.

### Lung cancer

RBM39 plays an important role in the occurrence and development of lung cancer. With its tumor-specific immune function, RBM39 has a unique role in regulating the biological functions of tumor cells [35, 42]. Kumar et al. confirmed that there were at least two RBM39 subtypes that could be used as tumor-associated antigens, which might stimulate the humoral immune response in human lung cancer patients [3]. Bangur et al. found 209 genes and nine initial characteristics of genes by identifying overexpressed genes in small-cell lung cancer, among which RBM39 was differentially expressed in non-small-cell lung cancer (NSCLC), which provided valuable basis for better understanding of the biological characteristics of NSCLC [42]. In order to further explore the role of RBM39 in the growth and migration of NSCLC cells, studies have shown that RBM39 was mainly located in the nucleus of lung cancer cells. After a series of analyses, it was found that the expression frequency of RBM39 in NSCLC tissues was significantly higher than that in adjacent tissues and normal tissues. Moreover, the overexpression of RBM39 could promote the proliferation and migration of NSCLC cells, and the expression of RBM39 had no obvious correlation with tumor stage, gender, and age, which preliminarily revealed that RBM39 was expected to become a biomarker and potential therapeutic target for lung cancer [3].

### Myeloma malignancy

As a new oncogene in myeloma, RBM39 is essential for the proliferation of multiple myeloma cells and tumorigenesis. Based on its biological role and clinical significance in tumor progression, RBM39 might become a key prognostic indicator for multiple myeloma patients [43]. Tong et al. implicated that RBM39 was highly expressed in myeloma cells, and its expression level was related to the poor prognosis of the tumor. Under hypoxia, knockout of RBM39 gene inhibited the mTOR signaling pathway, which could be reversed by RBM39 overexpression. In addition, further studies have shown that RBM39 inhibited the ubiquitination and degradation of RBM39 protein through the interaction with DARS-AS1, indicating that RBM39 might mediate the biological function of DARS-AS1 on multiple myeloma under hypoxic conditions [44]. Therefore, targeting the RBM39 axis might be a potential therapeutic target for multiple myeloma.

### Other cancers

According to previous studies, RBM39 is considered to be a novel antigen identified from patients with hepatocellular carcinoma, showing high levels of expression in hepatocellular carcinoma [1, 14]. It pointed out that anti-RBM39 autoantibody was the highest in liver cancer, and its immunostaining intensity in liver cancer cells was significantly reduced, and it was positively correlated with its decreased expression level, which further indicated that the decrease in RBM39 expression level might be related to the appearance of microvessels [45]. Additionally, RBM39 has also been reported in colorectal cancer and esophageal cancer. Anke H et al. found that RBM39 located on the 20Q-amplified fragment was upregulated in human colorectal adenoma and colorectal cancer, and affected cell survival and anchoring-independent growth by participating in some cancer-related biological processes [5]. Another study showed that the upregulated expression of RBM39 significantly increased the survival rate of colorectal cancer cells, suggesting that RBM39 has an extremely important potential function in apoptosis [46].

## Options modulating RBM39 expression in cancer

### Anticancer sulfonamides

**Fig. 3:**
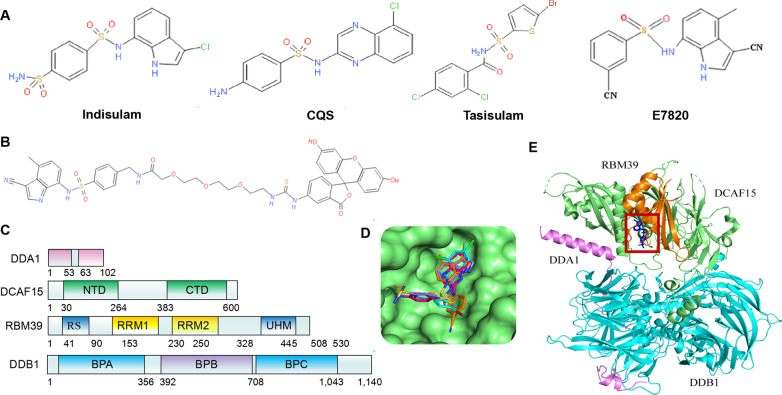
Structure of the complex of DDA1–DDB1–DCAF15–RBM39 with SPLAMs. **A** Chemical structures of indisulam, CQS, tasisulam and E7820. **B** Substitution reaction in the RBM39-indisulam interaction. **C** Domain representation of the proteins present in the complex. **D** The surface of DCAF15 is represented as green, and CQS, E7820, indisulam and tasisulam are represented as rods in red, orange, blue and cyan respectively. **E** Overall quaternary structure of human DCAF15–DDB1–DDA1–RBM39 (RRM2) in complex with aryl sulfonamides. DCAF15 is shown in green, DDB1 in blue, DDA1 in purple and RBM39 (RRM2) in orange. The aryl sulfonamide-binding site between DCAF15 and RBM39 is outlined in red.

**Fig. 4:**
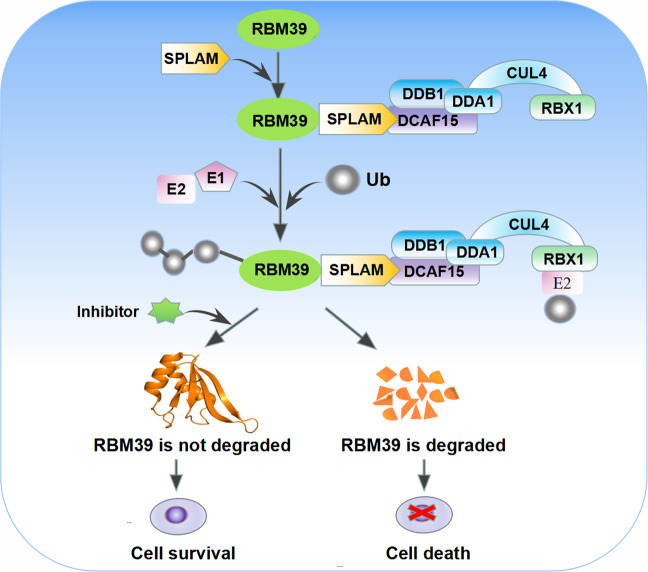
Schematic diagram of SPLAMs promoting degradation of RBM39. SPLAMs promote the interaction between RBM39 and DCAF15 E3 ligase substrate receptor, leading to ubiquitination of RBM39 and proteasome-mediated degradation, while proteasome inhibitors can prevent RBM39 degradation.

Recent studies revealed that SPLAMs, including indisulam, E7820, chloroquinoxaline sulfonamide (CQS), and tasisulam (Fig. 3A), are degradation products of RBM39, and DCAF15 was the main target of ubiquitination of SPLAMs [47, 48]. Therefore, SPLAMs could recruit splicing factor RBM39 to E3 ligase substrate receptor DCAF15, and promoted the proteasomal degradation of RBM39 through CRL4DCAF15-mediated ubiquitination, leading to splicing abnormalities of pre-mRNA dominated by intron retention and exon skipping [49] (Fig. 4). The main ubiquitination sites of RBM39 were located in the N-terminal region of 120 amino acids [29]. Moreover, SPLAMs used a mechanism similar to immunomodulatory drug (IMiDs) to target pre-mRNA splicing. IMiDs is an anticancer drug that could bind to the other adapter protein CRBN of CUL4 [47, 48]. It connected the endogenous E3 ubiquitin of CUL4/CRBN. Based on the inhibition of enzyme activity, other proteins were generalized into new substrates [48, 50]. However, studies found that the specific protein degradation of sulfonamide and lenalidomide was carried out independently, which led to clinically antimarrow tumor activity [29]. SPLAMs represent a new type of molecular gel-degrading agent after IMiDs. Specifically, they have a relatively weak receptor affinity [51]. After synergistically binding with DCAF15 and RBM39, they lead to the formation of a three-dimensional structure of DCAF15–DDB1–DDA1–RBM39 (RRM2), in which sulfonamides bind to the central helix of RBM39 (RRM2). It binds to DCAF15 (Fig. 3C, E), while DDA1 stabilizes the CRL4DCAF15 complex and promotes the recruitment of RBM39. In particular, RBM39 and indisulam had a substitution reaction during the interaction (Fig. 3B). We put the structures of the above-mentioned four related but structurally different sulfonamides in the same substrate receptor of DCAF15 (Fig. 3D) to further illustrate the structural similarity of sulfonamides. In addition, the four sulfonamides clinically tested showed the same mechanism of action [52]. The degradation of RBM39 was essential for anticancer activity, and the anticancer activity of SPLAMs was in direct proportion to the expression of DCAF15 and the dependence of RBM39 [52, 53]. Interestingly, the transcription of SPLAMs could downregulate RBM39, indicating that the expression of RBM39 might be negatively regulated by this protein [29]. In recent years, SPLAMs have been tested in phase II or III clinical trials in patients with metastatic cancer (Table 2), and have been found to progress relatively slowly in clinical trials due to their limited efficacy, with less than 15% of patients having a clinical response and an objective response rate of less than 40% in solid tumors [52]. The current clinical trials of SPLAMs mainly focus on leukemias and lymphomas that express DCAF15 protein at high levels, and have shown a certain degree of efficacy [54]. Therefore, the SPLAMs have potential clinical value in the treatment of cancer in the future, and they will also provide an important alternative splicing pathway opportunity for the growth of targeted cancer cells.

Indisulam (E7070) is a novel aryl sulfonamide with anticancer activity in vivo and in vitro discovered by Eisai pharmaceutical company in phenotype screening [55], and its antitumor activity in vivo is closely related to its effect on RBM39 [56]. With the increasing maturity of compound research, indisulam has shown effectiveness in a variety of clinical tumor models [57]. Moreover, it should be made clear that the anticancer agent indisulam is a target compound for the G1 phase of the cell cycle. As a carbonic anhydrase inhibitor, it inhibited the activation of CDK2 and cyclin E, causing the G1/S conversion to be blocked, and then the cells died [56]. Studies revealed that indisulam connected RBM39 protein to ubiquitin mechanism through the adapter protein DCAF15, thereby inducing the degradation of RBM39 and further inhibiting cell proliferation [53]. Therefore, the current tumor-targeted RNA splicing of indisulam is to recruit DCAF15 to induce the degradation of mRNA splicing factor RBM39 to target splicing [52]. At present, indisulam has conducted phase II clinical trials of patients with refractory or recurrent medullary malignancies, including indisulam combined with standard chemotherapy regimens, and has verified its clinical safety and disease stability [54, 58, 59]. More importantly, four phase I clinical trials have shown that the compound has nonlinear pharmacokinetics [60, 61]. At the same time, the compound is also undergoing phase II clinical trials in Europe and the United States, but the biological basis of indisulam sensitivity is currently unclear, so it has only shown limited efficacy in some patients [62, 63].

### Proteasome inhibitor

Targeted protein degradation is an emerging field in the development of small-molecule drugs. Cancer cells are highly sensitive to the cytotoxic effects of proteasome inhibition, making proteasome an important clinical target for targeted therapy of hematopoietic and lymphoid malignancies. As a kind of RNA-binding protein, RBM39 ubiquitination and proteasome degradation are essential for anticancer activity. Owing to its key role in the occurrence and maintenance of hematological malignancies, the degradation of proteasome has gradually become an important way for people to treat cancer. Nothing is more important than the fact that the reduction of RBM39 levels depends on the activity of the proteasome [52, 54]. Once it is degraded, it will lead to the abnormal splicing of pre-mRNA, which mainly includes intron retention and exon skipping, and leads to loss of cell viability and cell death [52]. At present, in the treatment of various hematological malignancies, a variety of small-molecule inhibitors have been developed and have successively entered the clinical trial stage (Table 3). Significantly, bortezomib, as the first proteasome inhibitor for the treatment of multiple myeloma, could block the indisulam-dependent degradation of RBM39 and showed better efficacy than other similar myeloma drugs [64]. It is considered that the approval of the drug set a precedent for the treatment of other malignant tumors [65]. Hereafter, bortezomib was used in combination with other drugs and found to have shown exciting results in the treatment of multiple myeloma and AML (Table 3). In addition, MLN4924 is a small-molecule inhibitor of UBA3, and it blocks the reduction of RBM39 induced by E7820 and the metabolism of Cullin RING ligases, so it is toxic to cells and is currently undergoing clinical trials [66]. It is reported that carfilzomib, a β5 inhibitor, was approved by the FDA for the treatment of multiple myeloma in 2012, and is currently undergoing phase I clinical trials as a potential PI for the treatment of leukemia [67]. Subsequently, the combination therapy of bortezomib and IMiDs provided a good prognosis for MM, and other proteasome inhibitors are undergoing clinical trials [68]. In general, target protein degradation is an emerging field in drug development and application. Anticancer sulfonamides and proteasome inhibitors can promote cancer progression by inducing changes in pre-mRNA splicing.

**Table 3:** Proteasome small molecule inhibitor of B5 protease for cancer treatment.

Modulators	Condition	Clinical Stage	Refs
Bortezomib + Daunorubicin + Cytarabine	Acute myeloid leukemia	Phase 1	[1]
Bortezomib + Decytabine	Acute myeloid leukemia, Myeloma malignancy	Phase 2	[2]
Bortezomib + SAHA		Phase 2 suspended	[3]
Bortezomib	Myeloma malignancy	Phase 1	[4]
Carlfizomib	Myeloma malignancy	Phase 1	[5]
Ixazomib	Myeloma malignancy	Phase 1	[6]
Marizomib	Myeloma malignancy, mantle cell lymphoma, chronic and acute lymphocytic leukemia, colorectal and pancreatic cancer models	Phase 3, recruiting	[7]
Delanzomib	Myeloma malignancy and other malignancies	Phase 1, Phase 2, terminated	[8]

## Concluding remarks

With the deepening of research, a growing number of scholars have generated a thought that RBM39, as a proto-oncogene, is not only closely related to the occurrence and development of a variety of malignant tumors, but also plays a crucial role in the clinical treatment of targeted drugs. As mentioned above, RBM39 indirectly participates in the growth and progression of tumors by regulating the transcription of many tumor-related genes, protein translation, and selective splicing, which provides new ideas and guiding significance for clarifying the pathogenesis of RBM39 in cancer. Since RBM39 was identified as the target of SPLAMs, the degradation of targeted proteins has gradually become an emerging field in the development of small-molecule drugs. It not only provides novel biomarkers for disease progression, but also affects the splicing of pre-mRNA subsets through the degradation of drugs. Therefore, CRISPR–Cas9-based DCAF15 gene knockout and single amino acid substitution of RBM39 can resist the degradation of RBM39 induced by sulfonamides, suggesting that RBM39 degradation is a key biochemical indicator of the anticancer properties of these compounds. At present, there are still many challenging problems to be solved. First, although the critical role of RBM39 in the occurrence and development of cancer has been supported by many reports, the current understanding of the mechanism of SPLAMs may be insufficient. Furthermore, although the safety of SPLAMs has been confirmed in many previous phase I and phase II clinical trials, it is still unclear whether RBM39 is an effective target of SPLAMs. Hence, it is necessary for future clinical studies to in-depth study the preferential effect of RBM39 degradation in cancer cells based on the spliceosome genotype, and to further determine the exact function of RBM39 in splicing. Although SPLAMs have only shown moderate efficacy in clinical trials so far, the biological basis of sensitivity to these drugs is still unclear, so they have only shown limited efficacy in some patients. Will SPLAMs be used in the treatment and research of more clinical patients in the future to improve the potential clinical efficacy of SPLAMs? Can SPLAM derivatives recruit new substrates other than RBM39 to join CUL4–DCAF15? Furthermore, so many questions are very necessary in the future research field to determine and further clarify the structural basis of CRL4–DCAF15 to identify sulfonamide degradation. The in-depth study of these sulfonamides may expand our understanding of the biological functions of RBM39, thereby guiding us to choose the correct target type that is more sensitive to these drugs for better application in cancer treatment. Taken together, further clinical trials will help determine the use of drugs targeting RBM39 activity in cancer therapy.

## Supplementary information

Abstract

cddiscovery-author-contribution-form
