# Supplementary material for: RNA-binding protein 39: a promising therapeutic target for cancer
Source: Cell Death Discov. 2021 Aug 13;7:214. doi: 10.1038/s41420-021-00598-7 (PMC8363639; doi:10.1038/s41420-021-00598-7)
Supplement: Supplementary file 1 — Abstract [file 41420_2021_598_MOESM1_ESM.docx]

**Abstract**

RNA binding motif protein 39 (RBM39), as a key factor in tumor-targeted mRNA and protein expression, not only plays a vital role in tumorigenesis, but also have broad development prospects in clinical treatment and drug research. Moreover, since RBM39 was identified as a target of sulfonamides, it has played a key role in the emerging field of molecule drug development. Hence, it is of great significance to study the interaction between RBM39 and tumors and the clinical application of drug targeted therapy. In this paper, we describe the possible multi-level regulation of RBM39, including gene transcription, protein translation and alternative splicing. Importantly, the molecular function of RBM39 as an important splicing factor in most common tumors is systematically outlined. Furthermore, we briefly introduce RBM39's tumor-targeted drug research and its clinical application, hoping to give reference significance for the molecular mechanism of RBM39 in tumors, and provide reliable ideas for in-depth research for future therapeutic strategies.
