# Supplementary material for: RNA-binding protein 39: a promising therapeutic target for cancer
Source: Cell Death Discov. 2021 Aug 13;7:214. doi: 10.1038/s41420-021-00598-7 (PMC8363639; doi:10.1038/s41420-021-00598-7)
Supplement: Supplementary file 2 — cddiscovery-author-contribution-form [file 41420_2021_598_MOESM2_ESM.pdf]

DECLARATION OF CONTRIBUTIONS TO ARTICLE

ADMC

Manuscript Number: CDDISCOVERY-20-1247-T

Journal Name: *Cell Death Discovery* (the 'Journal')

Proposed Title of the Contribution:

RNA-binding Protein 39: A Promising Therapeutic Target for Cancer (the 'Contribution)

Author(s):

Caipeng XU, Xiaohua Chen, Xuetian Zhang, Dapeng Zhao, Zhihui Dou, Xiaodong Xie, Hongyan Li, Hongying Yang, Qiang Li, Hong Zhang, Cuixia Di (the 'Authors')

For all *CDDis*covery articles, each person named as an author in the published version must be able to show he or she has contributed substantially to the article.

Authorship credit should be based on 1) substantial contributions to conception and design, acquisition of data, or analysis and interpretation of data; 2) drafting the article or revising it critically for important intellectual content; and 3) final approval of the version to be published. Authors should meet conditions 1, 2 and 3.

Any person who cannot be shown to have made a substantial contribution to the article cannot be listed as an author in the final version. The name of any person who is deemed to have made a minor contribution can, however, appear in the Acknowledgments section of the article.

Please complete the table below to indicate the contributions of all named authors to the manuscript.

Author Full Name:	Specification of Contribution to the Manuscript:
Qiang Li	Q Li meticulously conceived and designed the framework of the paper, and made strict revisions to part of the paper
Hong Zhang	H Zhang put forward reasonable opinions and suggestions on the important contents of the paper
Cuixia Di	CX Di designed the framework of this review, and made overall control and critical revision of the content in the paper
Xiaodong Xie	XD Xie gave key comments and suggestions on the content of the paper
Hongyan Li	HY Li gave some guidance to the content of the paper, the drawing of its tables and graphs, and put forward some guilding opinion
Hongying Yang	HY Yang made suggestions and new ideas for the research of RBM39
Caipeng Xu	CP Xu mainly wrote the paper
Xiaohua Chen	XH Chen further supplemented the paper
Zhihui Dou	ZH Dou was responsible for meticulously sorting out the relevant documents retrieved
Xuetian Zhang	XT Zhang made preliminary changes to part of the paper
Dapeng Zhao	DP Zhao was responsible for the retrieval of relevant documents

Please complete the table below to indicate the contributions of all named authors to the figures.

Figure 1:

CP Xu and XH Chen collected materials and information about this figure together, and finally CP Xu was responsible for drawing the figure. CX Di put forward some important opinions on the figure.

Figure 2:

DP Zhao was responsible for collecting the literature on the regulation mechanism of RBM39, and finally CP Xu drew a schematic diagram of the regulatory role of RBM39 in tumors, and Q Li provided comments and suggestions on the schematic diagram.

Figure 3:

The chemical structures of indisulam, CQS, tasisulam and E7820 were preliminarily drawn by XT Zhang. Finally, CP Xu was responsible for drawing the overall figure. H Zhang modified the schematic diagram.

Figure 4:

ZH Dou made a preliminary drawing of the schematic diagram of sulfonamides, and finally it was further drawn by HY Li. XD Xie and HY Yang put forward important opinions on the schematic diagram and made the final improvement.

Figure 5:

Figure 6:

Signed for and on behalf of the Author(s):	Print Name:	Date:
Cuixia Di	Cuixia Di	February 24,2021
